# The Effectiveness of Functional Exercises for Teaching Method Disaster Medicine to Medical Students

**DOI:** 10.7759/cureus.15151

**Published:** 2021-05-21

**Authors:** Wei-Kuo Chou, Ming-Tai Cheng, Chien-Hao Lin, Fuh-Yuan Shih

**Affiliations:** 1 Department of Emergency Medicine, National Taiwan University Hospital, Taipei, TWN

**Keywords:** disaster medicine, functional exercise, medical student, education, disaster exercise

## Abstract

Introduction

Functional exercises are effective for testing disaster management training. Previously, we found that functional exercises promote student engagement and improve the perception of learning after exercise.

Objective

The study objective is to investigate whether functional exercise is effective for teaching disaster medicine.

Methods

Students who partook in a two-day course of disaster medicine were recruited. The course consisted of lectures and workshops followed by a half-day functional exercise and was designed based on four core competency domains which included major disaster medicine concepts. After the lectures and workshops, participants completed a test to assess their knowledge of the core competency domains and a questionnaire to evaluate their willingness to pursue further training and participate in a disaster medical assistance team (DMAT) and their interest in disaster exercises. The functional exercise involved the scenario of an earthquake and mass-casualty incident and participants acted as DMAT members in the exercise. A post-exercise debrief was conducted by the evaluators to discuss performance and evaluate the results of the exercise. Participants then completed the same tests and questionnaires as before the exercise.

Results

Ninety-seven students were recruited, 72 of which were medical students. Pre- and post-exercise tests and questionnaires were completed by 48. We found disaster scene safety knowledge to be significantly improved after the functional exercise. Students’ willingness for further training and participation in a DMAT as well as their interest in disaster training was high before and after the exercise.

Conclusion

Disaster scene safety is a vital element of disaster medicine training but it is difficult to teach. Functional exercises represent a good tool for this purpose and can maintain enthusiasm for learning and participating in disaster medicine-related activities.

## Introduction

Disaster medicine is an important discipline for medical students. The Association of American Medical Colleges (AAMC) had suggested that training for this discipline should be included for all medical students [1,2], and previous studies have indicated the importance of such training [3]. Many disaster medicine or management courses have been investigated in previous studies, most of which have concluded that the current training is effective and receives good responses from students [3-16]. Didactic lectures, workshops, small group discussions, online video courses, tabletop exercises, and full-scale exercises have been discussed in past publications, although functional exercises (FE) are rarely used as a teaching method. In past experience, students who participate in FEs often find the exercise highly beneficial. Therefore, FEs are considered to be an effective method for teaching disaster medicine. The present study aimed to investigate which core competency of disaster medicine FE was most applicable for.

This article was previously presented as a meeting abstract at the 2019 International Conference on Emergency Medicine on June 12, 2019.

## Materials and methods

Study setting, design, and participants

A two-day course in disaster medicine was offered to all medical school students in Taiwan in May 2018. The first 1.5 days of the course consisted of lectures, workshops, and small group discussions. The competencies covered in this course were based on four core competencies for undergraduate college students (Table 1). The domains of each core competency were as follows: disaster patient care, disaster-scene safety, resource management, and communication. A 1.5-hour FE was conducted in the afternoon of the second and involved simulation of the scenarios of an earthquake and prehospital mass-casualty incident. The FE was designed based on topics taught in the first 1.5 days of the course, with the main purpose of practicing what students learned at this time. During the FE, students were asked to form a disaster medical assistance team (DMAT) and enact the treatment of multiple casualties at the scene. They were asked to assign themselves roles according to the Incident Command System (ICS). Patients, medications, and other resources were simulated using paper cards. Other simulations; including media, suspicious persons, town residents, government agency staff, search-and-rescue team, and DMAT hospital-based staff; were played by actors who were well trained for the exercise. Students were asked to organize triage and treat patients, communicate with local agencies and base hospitals, arrange patient transportation, manage medical and non-medical resources, speak at press conferences, and deal with suspicious persons and possible aftershocks. The entire exercise was observed by trained evaluators. A post-exercise debrief was conducted by the evaluators, who also provided feedback and lead discussions.

**Table 1:** The four core competency domains of the disaster medicine course.

Core competency domain	Core competencies
Disaster patient care	Demonstrate proficiency in the use of the mass-casualty triage system and proper application of medical care to patients
Disaster scene safety	Demonstrate proficiency in the recognition of disaster-scene safety issues
Resource management	Demonstrate proficiency in the management of medical and non-medical resources
Communication	Demonstrate proficiency in communication with media and other agencies

Measurements

All students who completed the exercise were asked to complete a questionnaire and test. Anonymous questionnaires were used to evaluate students’ willingness to pursue further disaster medicine training and participate in a DMAT, and to assess their interest in disaster training exercises. Students were asked to score each of these points using the 5-point Likert scale, where 1 = very low interest/willingness and 5 = very high interest/willingness. The test was designed based on previous research of learning-outcome evaluation, to evaluate students’ knowledge of the four disaster medicine core competency domains.7 The test included eight multiple-choice questions, using two questions to test each core competency domain. The pre- and post-exercise tests assessed the same core competency domains but utilized different questions to reduce repeat-testing bias [7]. The pre- and post-exercise tests also investigated students’ perception of learning in the exercise by asking students to rate this using the 5-point Likert scale (1 = very little and 5 = very much). Medical students who were involved in the whole exercise and completed both pre- and post-exercise questionnaires and tests were included in the study.

Analysis

Differences between pre- and post-exercise questionnaire scores were analyzed using a paired t-test. Pre- and post-exercise test scores for each core competency domain were analyzed separately from each other using a paired t-test. The significant level was set at 0.05 (two-tailed).

## Results

Characteristics of study subjects

Ninety-seven students attended the disaster medicine course. Among them, 72 were medical students and 48 of these completed the pre- and post-exercise questionnaires and tests. The response rate was 66.7%. First-grade students represented 41% of the cohort, and 87% had no previous disaster medicine training. Demographic data of participants are detailed in Table 2.

**Table 2:** Demographic information of the study population.

	Total number of participants = 48	
	Number of participants	Percentage
Grade		
First-year	20	41.67%
Second-year	5	10.42%
Third-year	8	16.67%
Fourth-year	4	8.33%
Fifth-year	4	8.33%
Sixth-year	5	10.42%
Seventh-year	2	4.17%
Number of previous training sessions		
None	42	87.50%
Once	5	10.42%
Twice	0	0.00%
More than three times	1	2.08%
Gender		
Male	22	45.83%
Female	26	54.17%

Main results

The scores of the pre- and post-exercise questionnaires are detailed in Table 3. Although the mean scores for each aspect of the questionnaire increased after the exercise, none of these differences were statistically significant. From the test results, the mean score for the core competency domains of disaster patient care and safety were significantly decreased and increased, respectively (Table 4). There were no statistically significant differences in the mean scores for the communication or resource management domains, although these scores increased and decreased slightly, respectively. Overall, the mean score of students’ perception of their learning from the exercise was 4.4. The distribution of this score is detailed in Table 5.

 

**Table 3:** Mean scores of the pre- and post-functional exercise questionnaires. Data are presented as mean (standard deviation). DMAT: disaster medical assistance team.

	Pre-exercise score	Post-exercise score	p-value
Willingness to pursue further disaster medicine training	4.3 (0.73)	4.6 (0.57)	p = 0.09
Willingness to participate in a DMAT	4.3 (0.75)	4.4 (0.61)	p = 0.65
Interest in disaster training exercises	4.3 (0.66)	4.5 (0.58)	p = 0.1

**Table 4:** Scores of the pre- and post-functional-exercise tests. Data are presented as mean (standard deviation).

Core competency domain	Pre-exercise score	Post-exercise score	p-value
Disaster patient care	1.83 (0.42)	1.45 (0.58)	p = 0.0005
Safety	1.22 (0.51)	1.91 (0.27)	p < 0.0001
Resource management	1.75 (0.43)	1.68 (0.51)	p = 0.52
Communication	1.72 (0.44)	1.83 (0.37)	p = 0.22

**Table 5:** Participants’ perceptions of learning scored using the 5-point Likert scale. From the Likert scale, learning was scored on a 5-point scale where 1 = very little and 5 = very much.

Perception of learning from the functional exercise	Number of participants	Percentage
1	1	2.08%
2	0	0.00%
3	2	4.17%
4	21	43.75%
5	24	50.00%

## Discussion

Disaster exercises are an important aspect of disaster preparedness and are generally classified into discussion-based and operation-based exercises [17]. Exercises are used to test capabilities, detect gaps, and train staff [10,16,18], especially in the case of operation-based exercises [19]. As an example of discussion-based exercises, tabletop exercises are commonly used in disaster preparedness training and education and have been reported on in a number of studies [4,8,9,12,20]. However, FEs - which are operation-based exercises - are not so commonly studied as a teaching method. A previous study has indicated that the utility of FEs for the education of health science students is insufficiently explored, but may have the potential [21]. Students partaking in the FE in the present study were usually highly involved compared with those participating in tabletop exercises in past experience. Therefore, it is reasonable to believe that FEs have potential as a teaching tool.

The high pre-exercise scores for willingness and interest noted in the present study are in line with the results of previous studies [22]. This may be because the attendance of the disaster medicine course and FE in the present study was voluntary. Another possible reason is that students felt that they had gained knowledge during the 1.5 days of the course and intended to apply what they just learned. The pre-exercise survey was not given prior to the course because we tried to focus on the effect of FE. The high pre-exercise scores may explain the lack of statistical significance in scores’ improvement after the exercise. However, increased mean scores of willingness and interest were noted after exercise despite the high pre-exercise scores. These results indicate that FEs can maintain students’ willingness and interest to learn rather than lower them due to frustration or pressure, which are frequently encountered in operation-based exercises. Most of the students in this study were junior medical students with no previous disaster medicine training. High willingness to pursue further learning and interest in disaster exercises may mean that students are more motivated to attend other training opportunities in the future. A high willingness to participate in a DMAT may indicate that more help can be expected when disasters occur. These findings demonstrate the effectiveness of FEs for maintaining medical students’ enthusiasm for disaster medicine.

Several explanations are proposed for these results. First, due to the nature of the course, students can apply what they have just learned from the lectures and workshops in the FE. This instant application will give them instant feedback and highlight where their knowledge or learning is inadequate. Debriefing after the exercise also provided feedback from the experts’ standpoints, providing further motivation to increase learning. Second, some of the potential difficulties and problems in DMAT work were disclosed to students in the exercise. The exercise scenario and injects were designed based on real events in history. Students were able to experience these difficulties and problems during the exercise and try to deal with them. This could give them the confidence to participate in a DMAT [13]. Third, the FE provides a strong impression of real-life disaster medicine. Previous research has indicated that a three-hour interactive tabletop exercise can be engaging for students and increase their knowledge [8]. Compared to tabletop exercises, FEs create an environment with higher tension, which may stimulate participation through the introduction of exercise injects and other participants. This can be fun and help to maintain students’ interest in the exercise.

Although disaster core competencies have been suggested as a scaffold for education and training [20,21], competency-based education is currently not common in medical schools [1] and few providers offer such training for a natural disaster or mass-casualty incident responses [14]. However, core competency-based approaches have been reported to be key for developing training exercises [23]. Based on these findings and suggestions [24], disaster core competencies were used to develop this disaster medicine course and FE. Several previous studies have proposed disaster core competencies [25,26] and indicated that students’ needs differ from those of other disaster practitioners [21]. Considering medical students with limited disaster response training [1] as the target audience, disaster patient care, safety, resource management, and communication were chosen as the core competency domains for this course and exercise [8,25,26]. Some of these have been highlighted as problems in disaster response [13], and they are topics that medical students should understand [12].

In order to design a course to teach these, several courses were evaluated for effectiveness. Most studies on competency-based education that report positive learning effects indicate the benefits of combinations of lectures and activities such as exercises. For example, Ingrassia et al used e-learning and classroom sessions involving problem-based learning activities, tabletop exercises, and computerized simulation to increase participants' knowledge of disaster medicine and basic competencies in mass-casualty triage [4]. Collander et al reported that a 2-day core competency-based disaster training course involving lectures and exercises increased healthcare workers’ knowledge, and high satisfaction was reported [9]. However, the teaching effects of disaster exercises are not fully understood, especially with regards to the core competency domains.

A review of the literature reveals that the most commonly reported focuses of patient care education are triage and first aid [2,27], with lectures and combinations of lectures and simulation exercises being the main methods to teach patient-care-related issues [2,13,27]. In the present study, these topics were also focused on as they were appropriate for the duration and type of exercise. However, the score for patient care given in the post-exercise test was lower than that of the pre-exercise test. This may be due to time constraints; the duration of the FE was 1.5 hours, and it was difficult to teach patient care in such a short time. A second possible explanation is that participants were mostly first-year medical students without clinical experience. This may influence their comprehension of patient-care-related issues [23]. Another possible explanation is the nature of the exercise. In this FE, patients were represented by paper cards with relevant information written on them. These may not provide sufficiently clear clinical impressions of patients, especially for students without clinical knowledge. These findings, therefore, suggest that FEs may not be suitable for teaching patient care to early medical students.

Even though it is frequently listed as a core competency domain of disaster training [3,9,25,26], safety rarely features in mass-casualty incident training [28]. However, it has been recommended that this topic is included in disaster training [29], and a combination of lectures and exercises is the typical teaching method [3,9]. This study showed a significant increase in the score that was given for safety in the test after the exercise. This may be because, in the FE, the safety issue was emphasized to students by injects, possibly causing a stronger impression of safety training compared with discussion-based exercises or lectures. A second reason could be that “role-playing” in the exercise may enhance the learning of this aspect [8]. Furthermore, a safe environment was provided in the FE. Full-scale exercise, another type of operation-based exercise, can provide the two effects of safety training mentioned above; however, students can experience the safety issue in FEs without actual danger, which may be encountered in full-scale exercises. Based on these results, FEs can be an effective tool to teach disaster-scene safety.

Resource management is an important core competency and is incorporated into training exercises in some universities [23]. This is generally taught via lectures or combinations of lectures and exercises [13,27]. The pre- and post-exercise scores for this domain were high in the present study, which may be the reason that a significant improvement was not observed after the exercise. Compared with lectures and discussion-based exercises, FEs can provide hands-on exposure to resource management, and these exercises are cheaper and easier to control than full-scale exercises. Therefore, FE is a useful approach for teaching resource management.

Communication has been suggested as a core competency domain of disaster medicine in previous studies, with the recommendation that this is taught to medical students [8,26]. A lecture-only approach reportedly failed to improve the knowledge of public health nurses with respect to communication [11], although it was reported to increase familiarity with the topic [27]. A combination of lectures and exercises has been shown to be more successful in improving knowledge and participant perception [3,9,13]. Tabletop exercises have been used as a sole method to teach communication issues, with successful increases in medical students’ knowledge reported [8]. The inclusion of communication as one of the core competency domains in full-scale exercises has also been studied [30]. Although the results lacked statistical significance, a slight increase in the test score for communication was noted after the exercise. The principles of communication are important to students, but it is often the case that real-life scenarios need to be encountered to really learn this skill. To this end, FEs provide opportunities for students to practice communication. In contrast to full-scale exercises, this FE was conducted in a classroom, which enabled evaluators to easily observe students’ performance and therefore provide more accurate feedback. Hence, FE can be an effective tool to teach and practice communication.

Most of the students in this study rated their perception of learning in the exercise as 4 or 5 out of 5, indicating that students felt they learned something beyond the core competencies. This may contribute to improving motivation to learn and participate.

Previous research has highlighted the benefits of competency-based education [24]. Despite the importance of core competencies in disaster medicine, few studies have discussed methods of teaching these competencies [26]. Using FEs as a teaching tool, teachers can apply this principle and teach the core competencies to students. The team who design the exercises can develop the scenarios and injects based on the particular core competencies that educators want to teach. This approach can also be used to further investigate the teaching effects in terms of each core competency.

Previous studies have demonstrated good results in either perception or test-score improvements, but have rarely discussed these with respect to specific core competency domains. This is, to the best of our knowledge, the first study to analyze the effects of a teaching tool in different core competency domains. This approach could be used to identify which specific teaching tool is most suitable for each core competency domain, and could be applied to other teaching modalities such as tabletop exercises, didactic lectures, and online video courses.

Limitations

Due to the course duration, time was limited and only two questions were tested for each core competency domain. Besides, the difficulty of pre-test and post-test might be different. Therefore, the statistical significance might be limited. Further large-scale studies involving more in-depth pre- and post-exercise tests should be considered.

Students had been subjected to several different education modalities prior to the exercise of this study, which may confound some of the teaching effects of the FE. However, the knowledge that was gained right before the exercise may have been further consolidated during operation-based exercises such as the FE.

The evaluation method of this study assessed students’ knowledge. An evaluation of the application of knowledge in the FE is not shown because the exercise was group-based instead of person-based. Further investigation of improvements in skills and other knowledge application following training should be carried out in the future.

## Conclusions

In summary, FE is not commonly used as a teaching modality in disaster medicine. However, the operation itself may be considered a method of learning. This study highlights that FEs can maintain the enthusiasm of medical students for further participation and learning, as well as being an effective tool for teaching safety in disaster medicine. This method of teaching can potentially be applied to other core competencies; in particular, issues that are vital but difficult to teach by lectures or tabletop exercises, such as coordination.
